# Human Fucci Pancreatic Beta Cell Lines: New Tools to Study Beta Cell Cycle and Terminal Differentiation

**DOI:** 10.1371/journal.pone.0108202

**Published:** 2014-09-26

**Authors:** Géraldine Carlier, Alicia Maugein, Corinne Cordier, Séverine Pechberty, Meriem Garfa-Traoré, Patrick Martin, Raphaël Scharfmann, Olivier Albagli

**Affiliations:** 1 INSERM U845, Research Center Growth and Signaling, Université Paris Descartes, Faculté de Médecine Cochin, Paris, France; 2 INSERM IFR94, Cytometry facility, Paris, France; 3 Endocells, Pépinière d’entreprises, Institut du Cerveau et de la Moelle Epinière, Paris, France; 4 IBDC - CNRS UMR 6543, Université Nice-Sophia Antipolis, Nice, France; Wayne State University, United States of America

## Abstract

Regulation of cell cycle in beta cells is poorly understood, especially in humans. We exploited here the recently described human pancreatic beta cell line EndoC-βH2 to set up experimental systems for cell cycle studies. We derived 2 populations from EndoC-βH2 cells that stably harbor the 2 genes encoding the Fucci fluorescent indicators of cell cycle, either from two vectors, or from a unique bicistronic vector. In proliferating non-synchronized cells, the 2 Fucci indicators revealed cells in the expected phases of cell cycle, with orange and green cells being in G1 and S/G2/M cells, respectively, and allowed the sorting of cells in different substeps of G1. The Fucci indicators also faithfully red out alterations in human beta cell proliferative activity since a mitogen-rich medium decreased the proportion of orange cells and inflated the green population, while reciprocal changes were observed when cells were induced to cease proliferation and increased expression of some beta cell genes. In the last situation, acquisition of a more differentiated beta cell phenotype correlates with an increased intensity in orange fluorescence. Hence Fucci beta cell lines provide new tools to address important questions regarding human beta cell cycle and differentiation.

## Introduction

The cell cycle consists in four phases: G1, S, G2 and M. In addition, in response to some situations (e.g. growth factor deprivation), cells can exit the cell cycle and reach the G0 phase primarily encountered in two cases: in quiescent stem cells, which can usually (re)enter the cell cycle upon appropriate stimulations, or in terminally differentiated cells, generally irreversibly withdrawn from the cell cycle [1]–[3].

Positioning cells within cell cycle at single cell or population level is the basis of cell cycle studies. However, the procedures dedicated to this aim are often time consuming, and generally destructive thereby precluding studies on live cells. Indeed, detection of markers used in cell cycle studies usually needs the fixation/permeabilization of the cells. While the staining of nucleic acids with some vital dyes is yet possible, it gives relatively imprecise information and is not suitable for all cell types [4]–[6].

Recently, several groups have designed new tools to conveniently define the position of fixed or living cells within the cell cycle. These new indicators are based on the constitutive expression of a gene encoding a chimeric marker, which consists in a fusion between a fluorescent protein and a cellular protein (or a part of it) that undergoes cell cycle regulation of its stability or distribution. Several new cell cycle indicators have thus emerged, using either proteins involved in DNA replication or in mitosis [7], [8]. To date, the most performant is the so-called Fucci system (Fluorescent Ubiquitination-based Cell Cycle Indicator). It combines two distinct fluorescent markers, namely human CDT1 (Cdc10 dependent transcript 1) fused to an orange fluorescent protein (monomeric Kusabira Orange, mKO2) and human GEMININ fused to a green fluorescent protein (monomeric Azami Green, mAG) [7]. Both CDT1 and GEMININ are direct substrate for distinct E3 ubiquitin ligase complexes, respectively SCF^skp2^ (Skp1-Cullin1-F-box protein) and APC^Cdh1^ (Anaphase Promoting Complex, also known as cyclosome), displaying mutual antagonism and hence reciprocal cell cycle-regulated activity [7], [9], [10]. Specifically,^.^ CDT1 protein is stable and accumulates during G1 but ubiquitinated for subsequent degradation by the SCF^skp2^ complex at the onset of S phase and thus absent throughout S/G2/M. GEMININ follows a symmetrical pattern: it is stable in S and G2, but targeted for ubiquitin-mediated proteolysis by the APC^Cdh1^ complex when cell exit mitosis and during G1. Each Fucci indicator consists of only a part of the wild type proteins (amino-acids 30–120 for CDT1 and 1–110 for GEMININ) designed to keep their susceptibility to cell cycle dependent regulation while minimizing their influence on cell cycle progression [7], [9]. Thus, in principle, the Fucci indicators allow the visualization of the major phases of the cell cycle (G1 cells are orange, S/G/M cells are green) but also the transitions since yellow (both orange and green) cells should correspond to early S cells and «black» (non fluorescent) cells are presumably in late M or early G1 [4], [7], [10].

The cell cycle of the pancreatic beta cells has been thoroughly investigated. However, despite these efforts, our knowledge of its regulation, especially in human, remains far from being complete. For instance, the mechanisms underlying the very slow turnover of beta cells after a perinatal wave of proliferation are poorly understood although age-dependent loss of responsiveness to PDGF probably partly accounts for this evolution [11]–[13]. In adult rodents, new beta cells arise primarily by duplication of preexisting beta cells while neogenesis (genesis of new beta cells from non-beta cells) mainly occurs before birth [14], [15]. In human, adult beta cells appear even more deeply resting, being probably mostly postmitotic and evidence for neogenesis is scarce [12], [16]–[18]. Hence, a number of questions remain unanswered: i) Why do young beta cells proliferate more than older ones? ii) Why do adult rodent beta cells proliferate more than human beta cells? iii) How beta cell mass homeostasis is achieved throughout human lifetime? Overall, the extreme paucity of *in* *vivo* observations in humans and the limited relevance of rodent models largely explain our poor current knowledge about human beta cell proliferation and call for new models to study its control [19].

*In* *vitro* studies on isolated human adult islets were also carried to provide insights into the control of the cell cycle. For instance, several cell cycle regulators or transcription factors, either alone or in combinations, appear to stimulate the proliferation of human adult beta cells [20]–[23]. However, these studies provide questionable conclusions as being mostly based on vast (adenovirus-mediated) overexpression. Moreover, standard proliferation markers *i.e.* BrdU incorporation and Ki67 expression were used in these studies. Yet, these markers may actually reveal an abortive cell cycle, presumably linked to DNA damages, in beta cells submitted to mitogenic stimulations, as in other terminally differentiated cell types [24], [25]. Accordingly, except in very few studies [26], whether induction of these proliferation markers is translated in an increased number of beta cells remains to be demonstrated [27]. Again, new methods and tools to study human beta proliferation would be highly desirable to overcome these limits. Toward this goal, we set up here human beta cell lines stably expressing the Fucci cell cycle indicators. We provide here several lines of evidence indicating that these new Fucci cell lines are reliable and convenient tools to decipher the control of cell cycle and differentiation of human pancreatic beta cells.

## Results

### EndoC-βH2 Fucci cell lines

Among various attempts to generate human pancreatic beta cell lines, the recently described EndoCβH1 cells offer the most compelling functional characterization [28]. This cell line was obtained by transduction of human fetal pancreatic cells with two lentiviral vectors encoding the large T antigen of the SV40 virus (SV40LT) and the human telomere reverse transcriptase enzyme (hTERT), both of them being controlled by the by the rat insulin promoter (RIP). To design human Fucci beta cells, we started from the closely related cell line, termed EndoC-βH2 [12] that has been generated in the same way as EndoCβH1, with one important difference providing more experimental flexibility: the two immortalizing transgenes, SV40LT and hTERT are flanked by Lox sequences. Consequently, they can be together removed upon expression of the Cre recombinase which induced the cells to cease proliferation and undergo a pronounced enhancement of β cell-specific features [28]. Thus, EndoCβH2 are extensively characterized human beta cells with a conditional immortalization and functional maturation.

**Figure 1:**
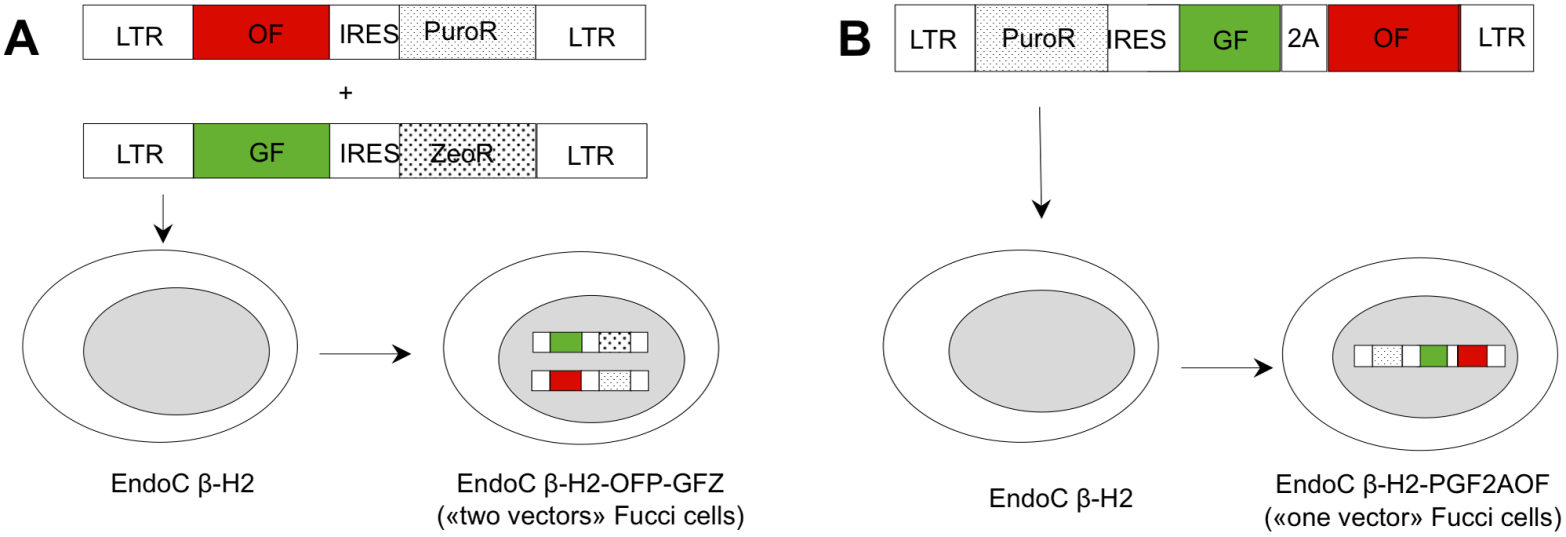
Generation of human beta Fucci cell lines. Two different EndoC-βH2 Fucci derivatives were generated. (A) In one EndoC-βH2 Fucci cell line, the two genes encoding the Fucci indicators (GF: green Fucci, mAG-ΔGeminin and OF: Orange Fucci, mKO2-ΔCdt1) were stably transferred by sequential retroviral transduction using two bicistronic vectors. They encode either GF plus the gene encoding the resistance to zeocin (ZeoR), or OF plus the gene encoding the resistance to puromycin (PuroR). After each step, a selection using the appropriate drug was achieved and the resulting cells line, termed EndoC-βH2-OFP-GFZ, was routinely cultured in presence of both selective drugs. (B) In the other EndoC-βH2 Fucci cell line, the two genes encoding the Fucci indicators (GF: green Fucci, eGFP-ΔGeminin and OF: Orange Fucci, mKO2-ΔCdt1) were present on the same retrovector and separated by a 2A peptide together with the gene encoding the resistance to puromycin. They were thus simultaneously transferred and the resulting cell line, termed EndoC-βH2-PGF2AOF, was selected and routinely cultured in a puromycin-containing medium.

**Figure 2:**
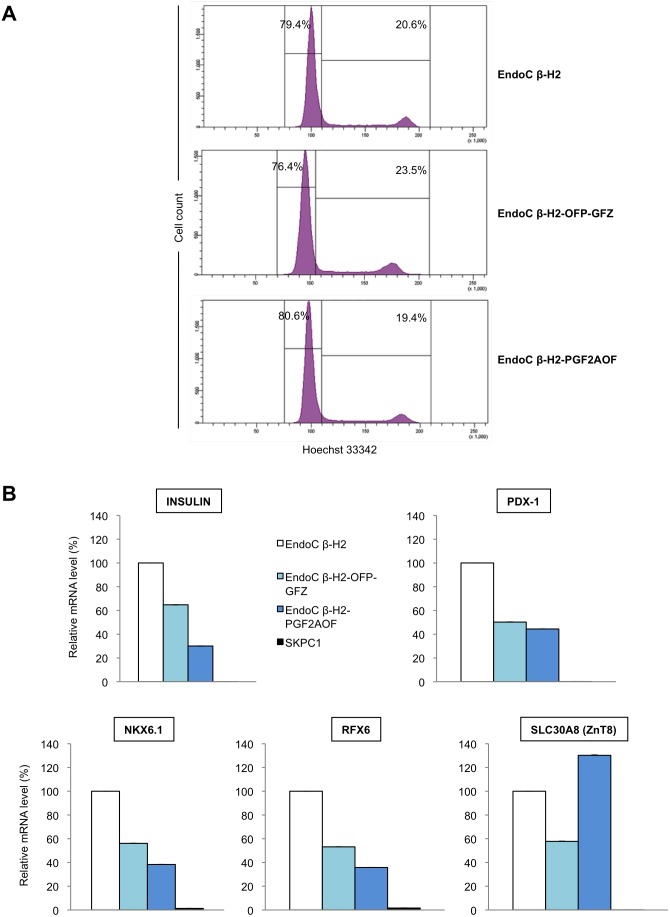
Introduction of the Fucci indicators does not alter the cell cycle nor the beta cell identity of EndoC-βH2 cells. (A) Exponentially growing EndoC-βH2 cells and their two Fucci derivatives EndoC-βH2-OFP-GFZ and EndoC-βH2-PGF2AOF were fixed, permeabilized and stained with the DNA dye Hoechst 33342 to be analyzed for position within cell cycle through their DNA content by flow cytometry. The three cell lines showed a very similar distribution of cells within cell cycle. (B) Expression of beta cell markers in EndoC-βH2 Fucci cells. The expression level of a selected set of genes encoding transcription factors (RFX6, PDX1, NKX6.1) or functional markers (INSULIN, SLC30A8/ZNT8) predominantly or exclusively expressed in beta cells was analyzed by quantitative RT-PCR in EndoC-βH2 cells and in their two Fucci derivatives, EndoC-βH2-OFP-GFZ and EndoC-βH2-PGF2AOF cells. Two PCR experiments, done with the same cDNA samples, were performed (two duplicates per sample), and the mean of the values as well as standard deviations are shown. For each transcript, mRNA levels were normalized to the highest values arbitrarily taken as 100.

We carried out two strategies to generate distinct Fucci EndoC-βH2 cell lines. In the first cell line, termed EndoC-βH2-OFP-GFZ, the two genes encoding the Fucci indicators (mKO2-ΔCDT1 or mAG-ΔGEMININ, thereafter referred to as Orange Fucci and Green Fucci, respectively) are encoded by two distinct retrovectors that were sequentially introduced. In the second one, termed EndoC-βH2-PGF2AOF, the Fucci indicators are encoded by a single retrovector and separated by the 2A «self-cleaving» peptide (Fig. 1) [29]–[31]. In this case, ΔGeminin is not fused to mAG (monomeric Azami Green), as in the EndoC-βH2-OFP-GFZ cells, but to the spectrally highly similar eGFP protein. Previous reports indicated that ΔGEMININ can be indifferently fused to many fluorescent proteins (including eGFP as well as several eGFP and DsRed derivatives) meaning that the physical turnover (emerging and degradation speed) of the S/G2/M Fucci indicator does not depend on intrinsic features of the fluorescent protein, but on the activity of the ubiquitin E3 ligase complex, APC^Cdh1^ [7], [9], [30]. The two resulting chimera, mAG-ΔGEMININ and eGFP-ΔGEMININ, can thus be regarded as functionally equivalent (as further shown below), and therefore, they are both referred to as Green Fucci thereafter. All vectors contain a selectable marker, and transductions were followed by a selection step to ensure that all cells harbor the retrovector. Importantly, stable introduction of transgenes encoding the Fucci reporters did not alter the cell cycle of EndoC-βH2 cells (Fig. 2A). Moreover, mRNA levels of several genes preferentially or exclusively expressed in beta cells (*INSULIN*, *NKX6.1*, *RFX6*, *PDX1* and *SLC30A8*/*ZnT8*) remained well above that observed in the human ductal cell line SKPC in both Fucci cell lines, although some of them appeared slightly reduced (1.3 to 2.5 fold in EndoC-βH2-OFP-GFZ cells, 2.5 to 3 fold in EndoC-βH2-PGF2AOF cells) when compared to parental EndoC-βH2 cells (Fig. 2B).

### Coincidence of Fucci markers with DNA content in EndoC-βH2 Fucci cells

**Figure 3:**
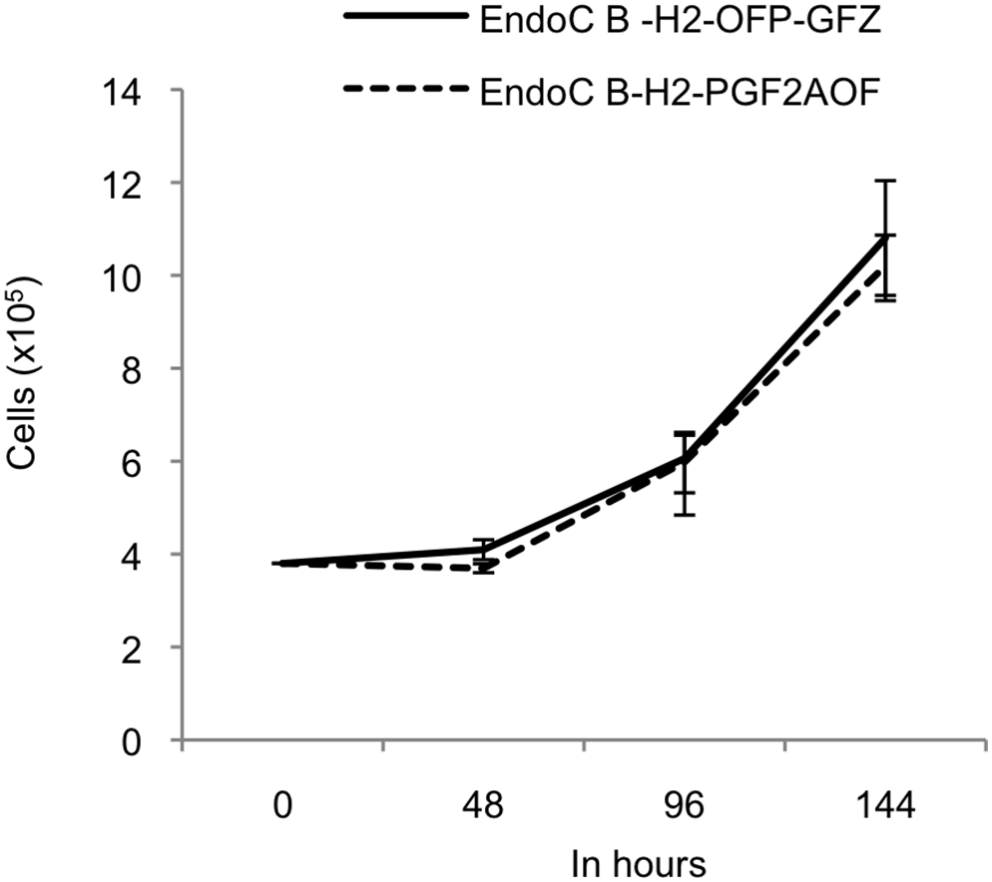
Doubling time of EndoC-βH2 Fucci cells. EndoC-βH2-OFP-GFZ and EndoC-βH2-PGF2AOF Fucci cells were plated at a density of 40×10^3^ cells/cm^2^ (0 h) in 6 well plates containing standard medium (without selection drugs), and the number of cells was measured and plotted at different time points. Each time point corresponds to the mean of the number of cells in four wells, and standard deviation is shown for each point. After about 5 days, the number of cells had approximately doubled in both cell lines.

**Figure 4:**
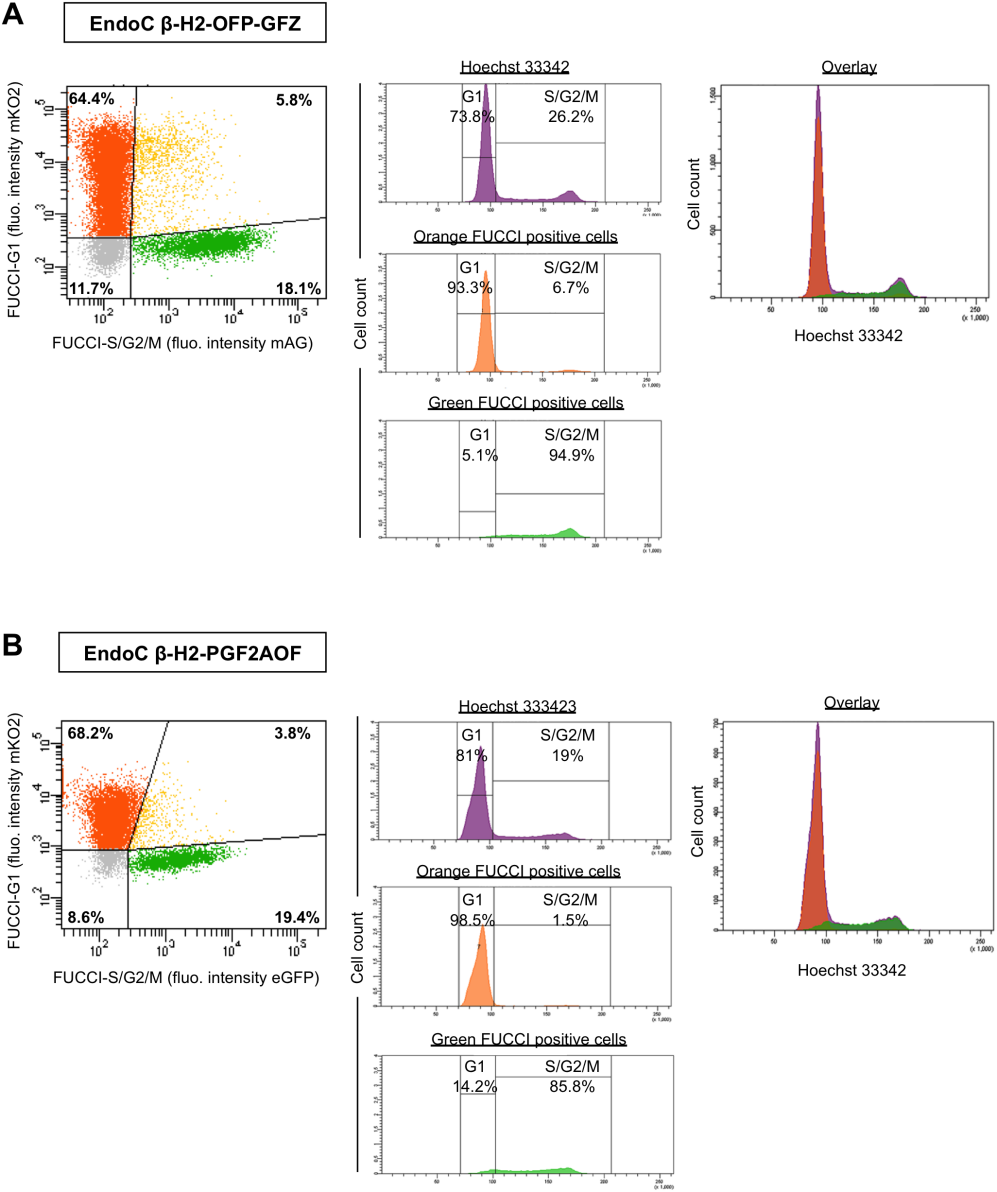
Comparison of DNA content and Fucci markers in human beta cells. (A) Exponentially growing EndoC-βH2-OFP-GFZ Fucci cells were fixed, permeabilized and stained with Hoechst 33342 for DNA content and analyzed for the three fluorescences (UV/blue for Hoechst 33342, green and orange for the two Fucci markers) by flow cytometry. Doublets were excluded from the analysis and the whole cell population subjected to the analysis is shown in violet (upper central panel). Its repartition thoughout the cell cycle can be compared with that of orange and green cell subpopulations (positive for the green and orange Fucci indicator, respectively) shown individually (middle and bottom central panels) or together (overlay, right panel). Left panel: cells were analyzed and shown according to their fluorescence. Percentages of green (right bottom area), orange (left upper area), yellow (green and orange, right upper area) and «black» (non fluorescent, left bottom area) are indicated. (B) Same as in *A* using EndoC-βH2-PGF2AOF cells.

Like parental EndoC-βH2 cells, both EndoC-βH2 Fucci cell lines are slow cycling, displaying a doubling time of about 5 days (Fig. 3) [28]. We examined the accuracy of the Fucci markers by measuring their degree of congruence with a third, independent, marker of the cell cycle. We stained exponentially growing, non-synchronized, EndoC-βH2-OFP-GFZ Fucci cells with Hoechst 33342, a dye revealing DNA content and well spectrally separated from the fluorescences emitted by both Fucci indicators. Hoechst 33342 staining indicated that most cells (76.4%) have a 2N DNA content, in agreement with their slow cycling rate (Fig. 2A). The thresholds for positive fluorescent signals were determined for each Fucci indicator using the parental EndoC-βH2 cells (doubly negative), as well as each simply positive control, namely EndoC-βH2 cells transduced with a retrovector encoding either Green Fucci (EndoC-βH2-GF) or Orange Fucci (EndoC-βH2-OP) (Fig. S1). Each of the two Fucci markers showed an almost complete coincidence with the cell subpopulation displaying the expected DNA content. Specifically, the vast majority (83%) of EndoC-βH2-OFP-GFZ Fucci cells having a 2N DNA content (G1 cells) were orange while, conversely, the vast majority (67%) of cells belonging to the S/G2/M population were green (Fig. 4A, middle panels). Similar results were obtained in EndoC-βH2-PGF2AOF cells (85% and 86%, respectively, Fig. 4B). Moreover, as previously reported in other experimental settings [7], [9], [29], time laps videomicroscopy experiments on both EndoC-βH2-OFP-GFZ and EndoC-βH2-PGF2AOF cells indicated that the green Fucci fluorescence vanished at this end of mitosis, leading to black (non fluorescent) cells for a few hours until the orange Fucci fluorescence became detectable in the two daughter cells (Fig. S2 and S3). We noticed that orange and green fluorescences are somewhat stronger in EndoC-βH2-OFP-GFZ cells than in EndoC-βH2-PGF2AOF cells (compare Fig. 4A and 4B) presumably because the genes encoding the Fucci indicators are located 5′ to the IRES element in EndoC-βH2-OFP-GFZ and 3′ to it in EndoC-βH2-PGF2AOF cells (Fig. 1) [14].

**Figure 5:**
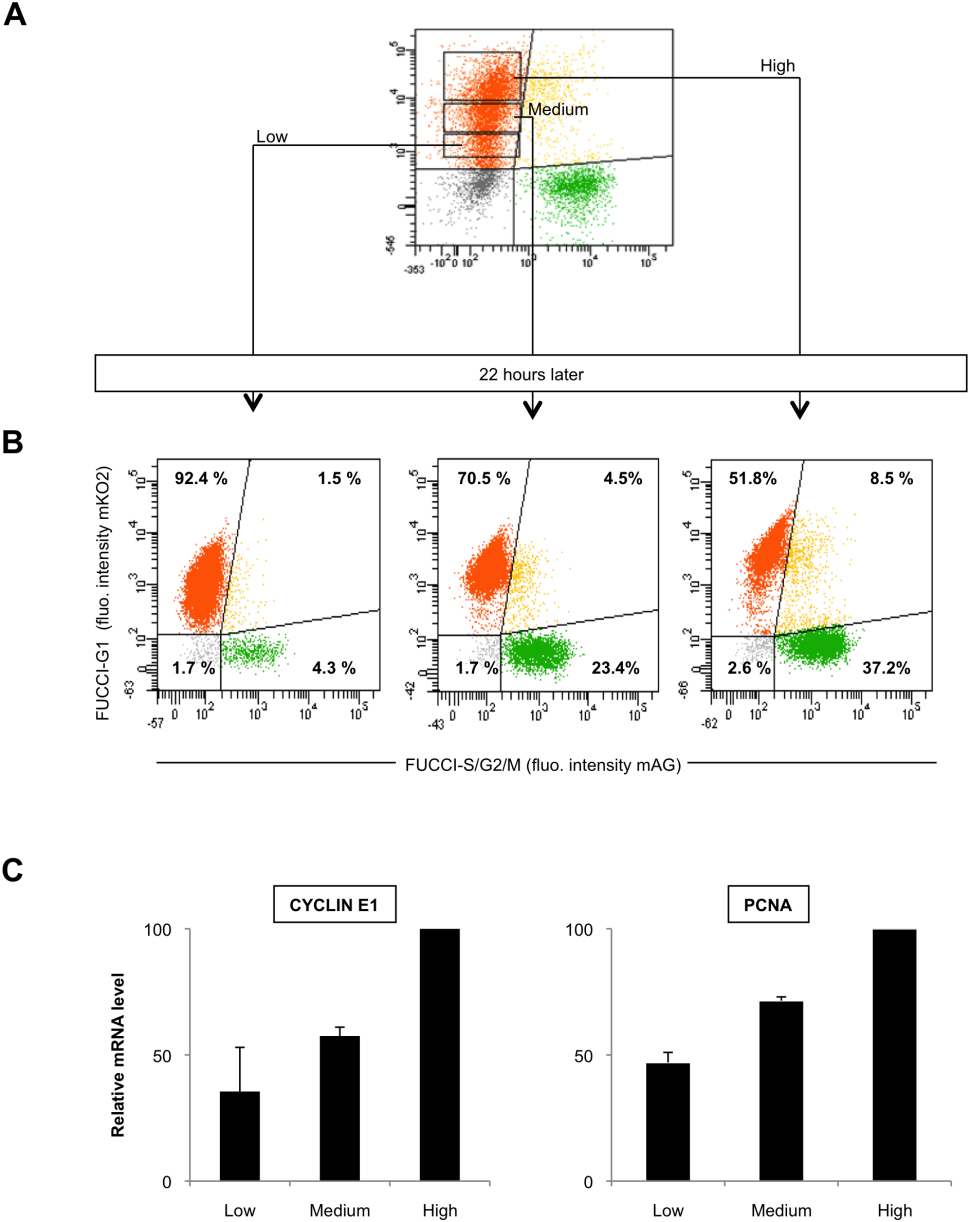
The intensity of the orange Fucci fluorescence is correlated with progression throughout G1 in human beta cells. (A) Exponentially growing EndoC-βH2-OFP-GFZ were FACS sorted according to the intensity of the orange Fucci fluorescence. Each sorted subpopulation (low, medium and high orange fluorescence) was immediately replated in separate wells. (B) 22 hours later, the cells in each well were fixed and analyzed by flow cytometry for its percentage of orange, green, yellow and «black» cells as in Fig. 4. Doublets were excluded from the analysis. The results shown in the three bottom panels indicate that the frequency of green and yellow cells in each population is strongly correlated with the intensity of the orange fluorescence of the fraction from which it derives. (C) Each of the three orange subpopulations sorted in A (low, medium and high) were analyzed for the mRNA levels of 2 late G1 markers, CYCLIN E1 and PCNA through quantitative RT-PCR analyses. The maximal value is arbitrarily taken as 100 for each transcript. Two PCR experiments, done with the same cDNA samples, were performed (two duplicates per sample), and the mean of the values as well as standard deviations are shown.

It has been reported that the intensity of the orange fluorescence reflects the time a given cell has already spent in G1 as the mKO2-ΔCdt1 protein progressively accumulates until the G1/S transition [7], [32]. We evaluated whether this correlation holds true for human beta cells. We FACS sorted and immediately replated three G1 subpopulations from growing EndoC-βH2-OFP-GFZ according to their level of orange fluorescence (low, medium and high) (Fig. 5A). Twenty two hours later, the percentage of green (S/G2/M) cells was 4.3%, 23.4% and 37.2% in cells derived from the low, medium and high orange fractions, respectively (Fig. 5B). This indicates that cells with the highest level of the orange fluorescence are more likely nearby the G1/S boundary. Confirming this conclusion, parallel quantitative RT-PCR analyses revealed that the low, medium and high orange fraction contained increasing amounts of PCNA (Proliferating Cell Nuclear Antigen) and Cyclin E1 mRNA, two markers of late G1 (Fig. 5C) [33], [34]. Therefore, EndoC-βH2 Fucci cell lines provide a mean to purify populations of living human beta cells enriched in cells in different sub-steps of G1. Note however that the high orange population is possibly not entirely made of G1 cells but may rather contain some early S phase cells which have not yet accumulated enough Green Fucci marker to be detected as positive for green fluorescence (see below).

### EdU staining and Fucci markers in EndoC-βH2 Fucci cells

The above-described results demonstrated a nearly perfect correlation between the Fucci indicators and DNA content. However cell cycle analysis by Hoechst 33342 staining is relatively imprecise. In addition, a small, albeit consistent, fraction of green cells was detected within the «G1 peak» (defined by Hoechst 33342 staining), most of them being thus yellow (double positive for green and orange Fucci) (Fig. 4, right panel). This could reveal some degree of leakiness of the green Fucci marker in human beta cells. Alternatively, these cells may be in early S phase with an increase in DNA over a 2N content too faint to be detected upon Hoechst 33342 staining, in line with the simultaneous expression of both Fucci indicators in cells just beginning DNA replication in other experimental systems [7]. In agreement with this possibility, time laps videomicroscopy experiments evidenced that the yellow fluorescence follows the orange fluorescence and precedes the green fluorescence in EndoC-βH2-OFP-GFZ cells (Fig. S4).

**Figure 6:**
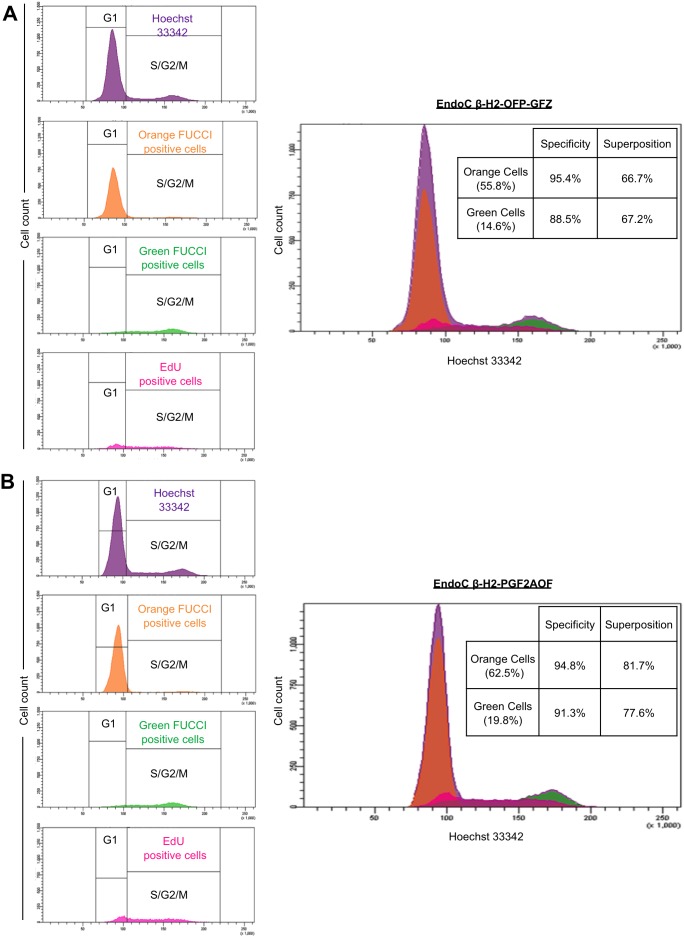
Comparison of ongoing DNA synthesis and Fucci markers in human beta cells. (A) Exponentially growing EndoC-βH2-OFP-GFZ Fucci cells were exposed for two hours to EdU, then fixed, stained with Hoechst 33342 for DNA content and analyzed by flow cytometry for four fluorescences (UV/blue for Hoechst 33342, green and orange for the two Fucci markers, far red for EdU). Doublets were excluded from the analyse. Left panels: each subpopulation emitting either orange (orange Fucci positive cells, in orange), green (green Fucci positive cells, in green) or far red (EdU positive cells, in pink) fluorescence is shown according to its DNA content (Hoechst 33342 staining) to be compared to each other as well as to the whole population (in violet). Right panel: overlay of the four fluorescences indicates. Almost all cells positive for both Fucci markers in the «G1 peak» are in S phase (positive for EdU). Based on this four color analysis, two ratio were defined to show the accuracy of the Fucci indicators. First the «specificity rate», which means among orange (green) cells, the percentage of cells being in G1 (S/G2/M) based on both Hoechst 33342 and EdU stainings. Reciprocally, the «superposition rate» corresponds to the percentage of orange (green) cells among G1 (S/G2/M) cells. The percentages indicated in the first column corresponds to those in the whole cell population. Note that the fluorescence intensity is somewhat decreased by the protocole used to reveal EdU which lowers the «superposition rate» compared to that reached under standard conditions (see Fig. 4). (B) Comparison of ongoing DNA synthesis and Fucci markers in EndoC-βH2-PGF2AOF cells. Same as in A using EndoC-βH2-PGF2AOF cells.

To discriminate between these possibilities, we performed a four-colors flow cytometry analysis by adding to the three fluorescent markers described above, the thymidine analogue EdU revealed by a far-red fluorochrome. EdU, which is metabolically incorporated in DNA of all S phase cells, revealed that the «G1 peak» (defined by Hoechst 33342 staining) contained a small proportion of S phase cells that were also positive for the green Fucci marker (Fig. 6A and 6B, left panels). Hence, the green Fucci marker is highly specific of S/G2M phase in beta cells, and yellow cells defined by flow cytometry are indeed in early S. Interestingly, while EdU signal was detected in almost all double positive (yellow) cells, it also stained rare orange-positive/green-negative beta cells within the «G1 peak» (Fig. 6A and 6B, left panels). This presumably results from a short lag existing between S phase entry and both orange Fucci complete proteolysis and green Fucci fluorescent maturation [7]. This lag appears comparable in the two Fucci cell lines, consistent with the rapid maturation (« ripening ») of both eGFP and mAG [35]. Of note, EdU positive cells are also detected in the G2/M peak which itself accounts for approximately 10% of the analyzed population in both EndoC-βH2-OFP-GFZ and EndoC-βH2-PGF2AOF cells. This is presumably because they have progressed from S to G2/M during the pulse. As this pulse lasted for 2 hours, and given about 20% of EdU cells are in G2/M (527/2141 for EndoC-βH2-OFP-GFZ cells, 835/4188 for EndoC-βH2-PGF2AOF cells), we can incidentally estimate the duration of the S phase to about 10 hours.

Importantly, according to this four color analysis, the Fucci reporters were confirmed to be highly reliable in both EndoC-βH2-PGF2AOF and EndoC-βH2-OFP-GFZ cells. Indeed, about 95% of orange cells were found in G1 and nearly 90% of green cells were in S/G2/M, based on Hoechst 33342 and EdU stainings («specificity rate», Fig. 6 right panels). Reciprocally, in both cell lines, the vast majority of cells in G1 and in S/G2/M were orange and green, respectively («superposition rate», Fig. 6 right panels). Thus, the Fucci indicators faithfully read-out the position of human beta cells within the cell cycle. Noteworthy, the reliability of the Fucci indicators is weaker when they were individually introduced in EndoC-βH2 cells (Fig. S1), suggesting that their concomitant expression underlies in part their mutual exclusion and minimal toxicity throughout the cell cycle [7], [36].

### The Fucci indicators reflect modulation in EndoC-βH2 proliferation

**Figure 7:**
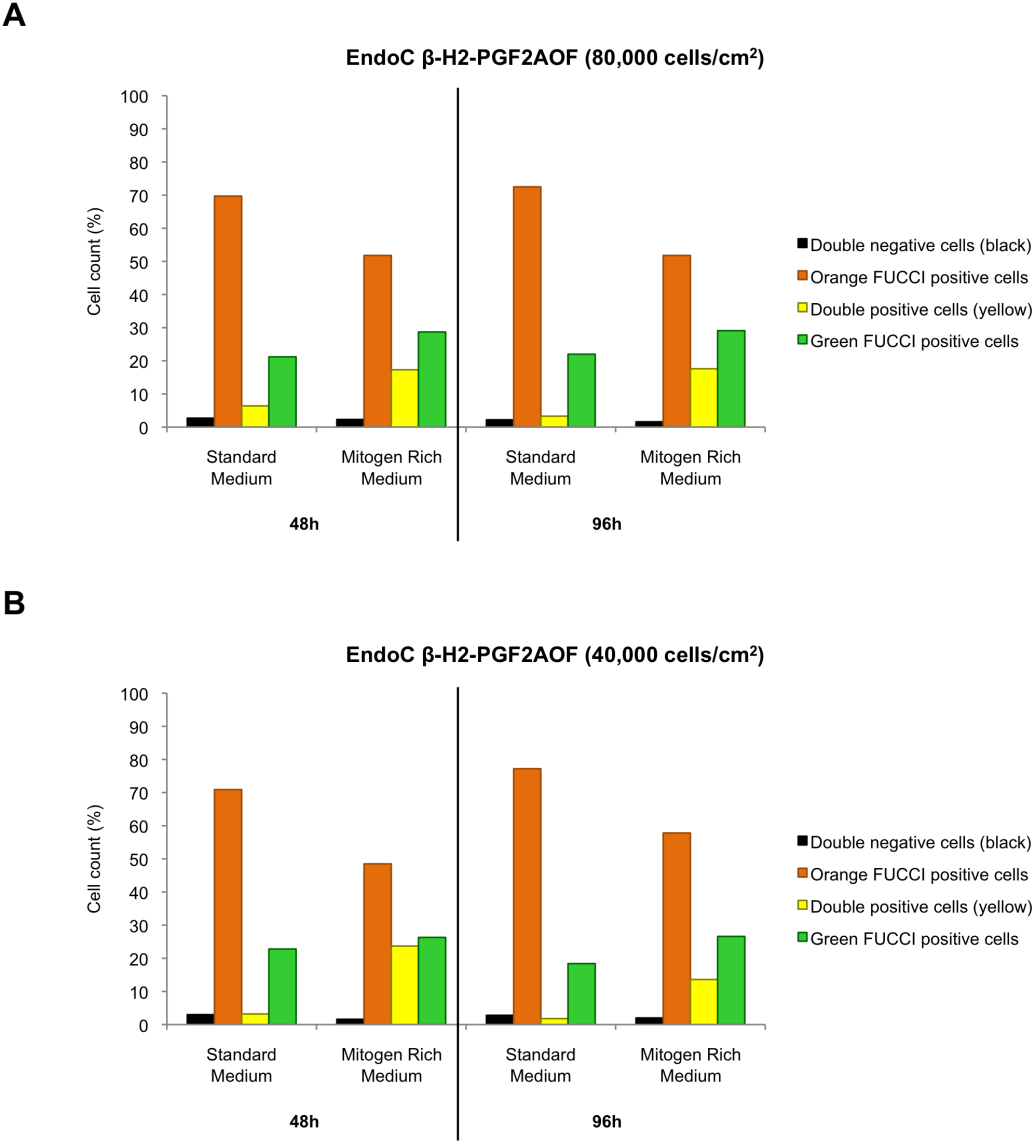
Fucci indicators reveal the exposure of human beta cells to a mitogen-rich medium. Exponentially growing EndoC-βH2-PGF2AOF Fucci cells were seeded at either 80×10^3^ cells/cm^2^ (A) or 40×10^3^ cells/cm^2^ (B) in either standard medium or a mitogen-rich medium and analyzed for green and orange fluorescences through flow cytometry after 48 hours (left parts of the panels) or 96 hours (right parts of the panels). Doublets were excluded from the analysis. The percentages of cells displaying green, orange, «yellow» (double positive) or no fluorescence (« black », double negative) were plotted for each culture condition and time point.

The above-described experiments were performed on exponentially growing beta cells randomly positioned throughout the cell cycle. To check whether the Fucci markers could be also used to screen for signals that modulate beta cell cycle, we exposed Fucci beta cells seeded at the usual density (80×10^3^ cells/cm^2^) to a mitogen-rich medium. After both 48 h and 96 h, this mitogenic stimulation of EndoC-βH2-PGF2AOF cells increased the proportion of green (S/G2/M) cells (from 21.2% to 28.7% after 48 h, from 22% to 29.1% after 96 h) while decreasing the orange (G1) population (from 69.7% to 51.8% after 48 h, from 72.5% to 51.8% after 96 h) (Fig. 7A). The yellow population (positive for both orange and green Fucci) revealing the early S cells was also markedly expanded under these conditions (from 6.4% to 17.4% after 48 h, from 3.3% to 17.6% after 96 h). Very similar results were obtained when EndoC-βH2-PGF2AOF were seeded at a lower density (40×10^3^ cells/cm^2^) and submitted to the same culture conditions (Fig. 7B).

**Figure 8:**
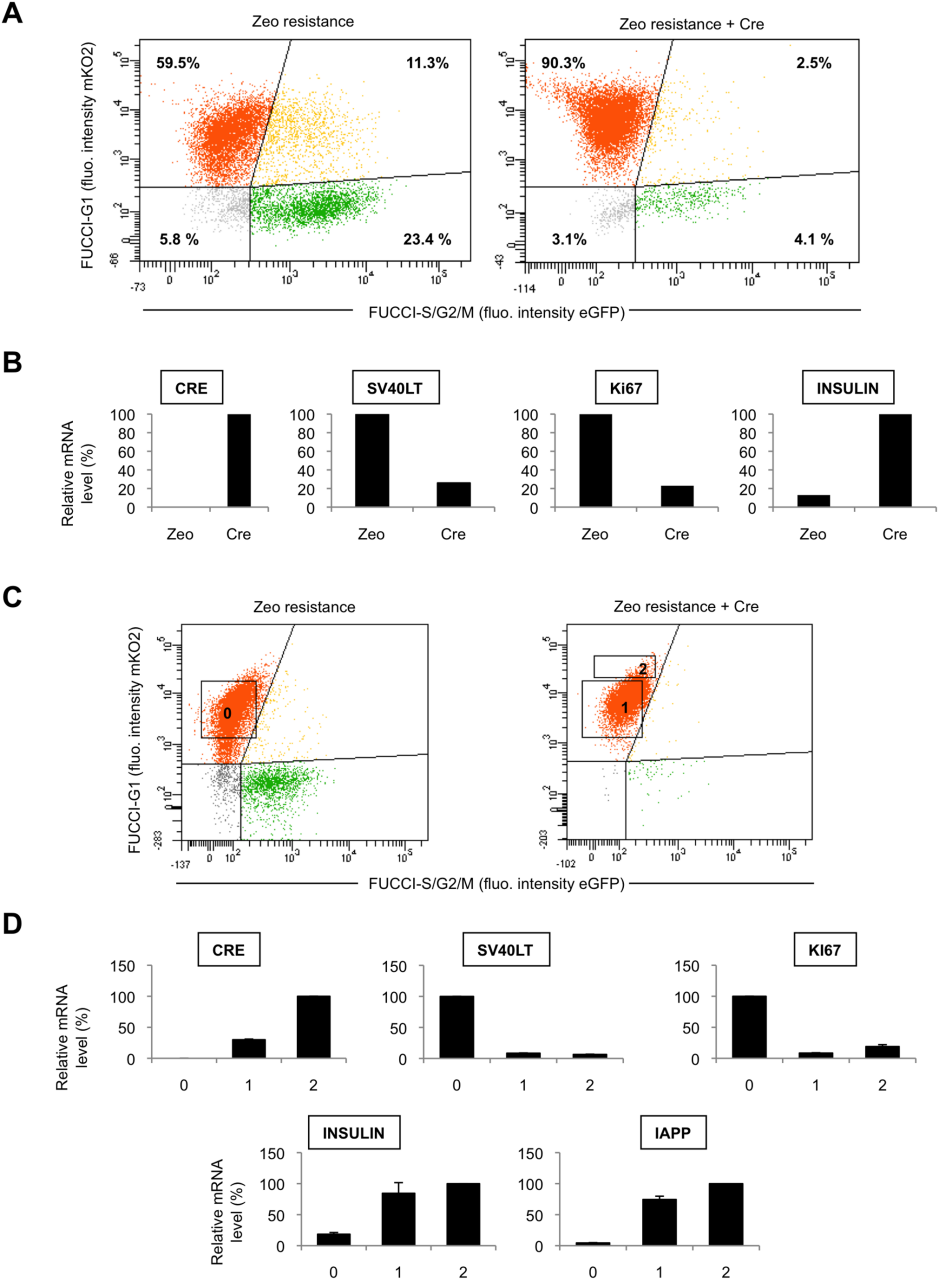
Fucci indicators reveal growth-arrest and terminal differentiation of human beta cells. (A) Exponentially growing EndoC-βH2-PGF2AOF Fucci cells were transduced with a retrovector encoding either a selectable marker (zeocin resistance) or both Zeocin resistance and the Cre recombinase, then selected in a zeocin containing medium and analyzed by flow cytometry for their green and orange fluorescences 13 days later. Doublets were excluded from the analysis. EndoC-βH2 cells depends upon two floxed transgenes for their proliferation, *SV40LT* and *hTERT*. After 13 days (time of transduction taken as day 0), green EndoC-βH2-PGF2AOF cells become much rarer while the proportion of orange cells inflates in cells transduced with a Cre-encoding vector (right) compared to control cells (left). (B) Control and Cre-transduced cells were analyzed for expression of *Cre*, *SV40LT, Ki67* and *INSULIN* mRNA by quantitative RT-PCR. The maximal value is arbitrarily taken as 100 for each transcript (C) The same experiment as in A was carried out and control and Cre-transduced cells were FACS sorted after 12 days (time of transduction taken as day 0) according to the intensity of the orange fluorescence: the bulk of orange control cells (fraction 0), the exactly corresponding fraction among CRE-transduced orange cells (fraction 1) and the brightest Cre-transduced orange cells (fraction 2). (D) Each fraction harvested in (C) was then submitted to quantitative RT-PCR analyses for the expression of *Cre*, *SV40LT, Ki67*, *INSULIN* and *IAPP* mRNA. Two PCR experiments (except three for Insulin) done with the same cDNA samples, were performed (two duplicates per sample), and the mean of the values as well as standard deviations are shown. The maximal value is arbitrarily taken as 100 for each transcript (bottom panels).

To examine whether symmetrical effects are observed when proliferation is shut down, we took advantage on distinctive features of EndoC-βH2 cells. Overall, EndoC-βH2 cells appear as relatively immature beta cells whose phenotype (slow proliferation, suboptimal expression of several beta cell markers) has been reversibly frozen by the two floxed immortalizing transgenes, SV40LT and hTERT. Upon removal of these transgenes, EndoC-βH2 cease to proliferate, exit from cell cycle and further increase expression of several beta cell specific genes, including insulin, within 10–20 days [12]. We thus transduced EndoC-βH2-PGF2AOF cells with a Cre-encoding retrovector that harbors a selection marker, the zeocin resistance to eliminate the non-transduced cells. Thirteen days after transduction, the proportion of orange cells dramatically increased compared to control cells transduced with a retrovector only encoding the zeocin resistance gene: orange cells accounted for 59% in control cells but for more than 90% in Cre-expressing cells while the frequency of green and yellow cells concomitantly dropped (Fig. 8A). Quantitative RT-PCR experiments showed that expression of the proliferation marker *Ki67* sharply decreased while that of *INSULIN* increased upon Cre expression (Fig. 8B).

Interestingly, orange Cre-expressing cells are overall more fluorescent than their control counterparts. Specifically, in Cre-expressing cells, barely fluorescent orange cells almost completely vanished while a very bright fraction inflated (Fig. 8C, compare left and right panels). Starting again from the assumption that the intensity of orange Fucci is a «clock» measuring the length of G1 phase, we wondered whether such very bright orange fraction corresponds to cells more advanced in their differentiation, a process generally associated with G1 lenghtening [2], [3]. EndoC-βH2-PGF2AOF cells were exposed once again to either Cre-encoding or control retrovector and after 12 days three fractions of orange cells were FACS sorted for quantitative RT-PCR analyses: the bulk of the orange population in control cells, their exact counterparts among Cre-expressing cells and the very bright (Cre-expressing) orange cells (Fig. 8C). While both Cre-expressing fractions displayed a similar reduction in *SV40LT* and *Ki67* mRNA levels (compared to control orange cells), the very bright orange cells express higher mRNA levels of two markers of mature beta cells, insulin and IAPP (Islet Amyloid PolyPeptide, or Amylin), correlating with a stronger Cre expression (Fig. 8D). We conclude that the intensity of the orange Fucci marker reflects the degree of beta cell terminal differentiation upon excision of immortalizing transgenes.

## Discussion

In this work, we engineered EndoC-βH2 cells, which results from targeted and reversible immortalization of human fetal pancreatic cells, to derive beta cell lines stably expressing the Fucci cell cycle indicators. Flow cytometry analyses on exponentially growing, non-synchronized, EndoC-βH2 cells showed that the type of the Fucci fluorescence emitted by the cells reveal their position into the cell cycle: orange cells and green cells are almost perfectly coincident with G1 and S/G2/M cells. Moreover, two major transitions of the cell cycle of human beta cells are also revealed by our Fucci cell lines since yellow cells (positive for both Fucci markers) are in early S phase while « black » cells (negative for both Fucci markers) are in late M or early G1. Finally, the intensity of the Fucci marker fluorescences also gives useful informations as it provides an easy mean to detect (and purify) cells from more precise « sub-phases» of the cell cycle. For instance, early and late G1 human embryonic stem cells were recently sorted on the basis of the intensity of the orange Fucci fluorescence [32]. Similarly, we show here that the increase in the intensity of the orange Fucci marker correlates with the likelihood of the cells to be close to the G1/S boundary. Thus, our Fucci cell lines provide a way to study human beta cell progression throughout G1 (through transcriptomic or proteomic analyses of sorted cells) which represents, under our conditions, the largest part of their cell cycle and therefore the major cause of their slow expansion, at least *in* *vitro*.

The Fucci markers also faithfully reflect global changes in the proliferation of cultured human beta cells. Indeed upon exposure to a mitogenic-rich medium, the number of green and yellow cells increases at the expense of the orange cells. Conversely, Cre-mediated withdrawal of immortalizing transgenes (*SV40LT* and *hTERT*) is accompanied by reciprocal changes in green, yellow and orange cell populations. Altogether, these data indicate that the Fucci indicators fluctuated as expected when human beta cells undergo modulation, either positive or negative, in their proliferative activity. EndoC-βH2 Fucci cells are thus perfectly suitable for high throughput screening of chemicals modulators of the cell cycle.

How immature human beta cell to exit from the cell cycle and terminally differentiate is poorly understood. EndoC-βH2 cells provide a unique tool to open this black box as these events are inducible upon EndoC-βH2 transduction with a Cre-encoding vector [28]. We show here that EndoC-βH2 Fucci derivatives should give additional grips to further experimentally assess these events. In some stem cells/progenitors, such as mouse neural stem cells/progenitors or human embryonic stem cells, an increasing intensity of the orange Fucci indicator reflects ongoing differentiation most probably because both events are linked to G1 lengthening [2], [37], [38]. In the same way, FACS sorting experiments revealed that the mRNA levels of two markers of mature beta cells, *INSULIN* and *IAPP*, are higher in the brightest orange Cre-transduced EndoC-βH2 Fucci sub-population than in other orange Cre-transduced cells and, to a much greater extent, than in control (Zeo-transduced) orange cells. Meanwhile, expression of *SV40LT* and of *Ki67* is similarly reduced in both Cre-transduced populations. In summary, control orange cells can be seen as immature beta cells in G1 (*INSULIN*/*IAPP* low, *Ki67* high) while both the two orange populations among Cre-transduced cells are in G0 (*Ki67* low) but displayed distinct degree of terminal differentiation (*INSULIN/IAPP* medium-high or high). Therefore, the exit from cell cycle and terminal differentiation can be monitored and separately analyzed by following-up the intensity of the orange fluorescence in Cre-transduced EndoC-βH2 Fucci cells. Combined with cell sorting, this provides a mean to better understand each step leading to the acquisition of a fully matured phenotype in cultured human beta cells. Moreover, the green Fucci marker can be potentially used to study if the exit from cell cycle of Cre-transduced EndoC-βH2 Fucci cells can be made reversible as a (hypothetical) re-entry of these cells into the cell cycle should reawaken this fluorescence. In this way, a sensitive screening can be set up to test factors (either soluble or endogenous) that might modulate human beta cell proliferation, as well as to study whether, or not, a restart of EndoC-βH2 Fucci proliferation would be accompanied by a partial de-differentiation. Altogether, EndoC-βH2 Fucci cell lines appear as promising tools to address important questions regarding human beta cell cycle and differentiation.

## Experimental Procedures

### Cell culture

EndoC-βH2 cells have been extensively described in ref [28]. They were cultured on Matrigel (1.2%) and fibronectin (3 µg/ml) (Sigma-Aldrich)-coated dishes in the following medium: DMEM containing 5.6 mM glucose, 2% bovine fatty acid free serum albumin fraction V (BSA, Roche diagnostics), 50 µM 2-mercaptoethanol, 10 mM nicotinamide (Calbiochem), 5.5 µg/ml transferrin (Sigma-Aldrich), 6.7 ng/ml selenite (Sigma-Aldrich), 100 U/ml penicillin, and 100 µg/ml streptomycin [28]. Appropriate drugs were added to select and culture transduced cells (see below). Every 5 days, cells were passaged and replated in 2 identical tissue culture plates. Unless otherwise stated, they were plated at a density of 80×10^3^ cells/cm^2^. The mitogen-rich medium is derived from the one recently used for pancreatic cells [39].

### Vectors

All retrovector constructs were derived from the pPRIG retrovector or its relatives [40]. Two new pPRIG derivatives were used in this work: pPRiHy and pPRIZ vectors in which the eGFP cDNA in pPRIG was replaced by the Hygromycin- or Zeocin-resistance gene, respectively. mCAT1 cDNA was recovered from a lentiviral vector constructed by the Shinya Yamanaka’s lab (Kyoto University, Japan), and purchased from Addgene (pLenti UbC mSlc7a1, Plasmid #17224). mCAT1-HA cDNA was recovered from a pTarget mCAT-HA constructed by Yoshinao Kubo (Nagasaki University) provided by Sébastien Storck (INSERM U783, Paris, France). The Cre-encoding retrovector harbors a Cre-HA cDNA designed by modifying a Cre-GFP cDNA from Connie Cepko lab (Harvard Medical School, Boston, USA) and purchased from Addgene (pCAG Cre-GFP, Plasmid #13776).

### Cloning strategy and generation of the expression vectors

#### mCAT1 and mCAT1-HA encoding vectors

pCMVmCAT used to produce mCAT gesicles was constructed in two steps: first, the eGFP cDNA was removed from the peGFP-C1 vector (Clontech) by a Eco47III and Ecl136II digestion, followed by religation. The resulting vector was then digested by EcoRI and SalI to be ligated with the mCAT-1 cDNA digested by EcoRI and XhoI from the pLenti UbC mSlc7a1. pTarget mCAT1-HA was digested by XhoI and NotI cloned into the pPRIHy retroviral vector digested by the same enzymes.

#### Fucci encoding retrovectors

The gene encoding the orange Fucci or green Fucci indicator was taken by digesting the pFucci-G1 orange vector or pFucci-S/G2/M green vector (both of them purchased from MBL Life Sciences) by BamHI and SphI and the cloned into the pPRIPu or pPRIZ retroviral vector, respectively, digested by the same enzymes. The retrovector encoding the two Fucci indicator has been constructed by starting with a 1497 bp DNA fragment synthesized and cloned into a SacI and PstI digested pBluescript by Epoch Biolabs, INC. (1306 FM1092 Rd, Ste 407 Missouri city, TX 77459-1565) generating the pBSK Ucci vector. This 1497 bp fragment was engineered to express a fusion protein of the following domains in this order: ΔGEMININ, a 2A peptide, the mKO2 fluorescent protein and ΔCDT1. A BstXI-Bsp1407I fragment eGFP encoding from the pPRIG vector was then cloned into the pBSK Ucci digested by the same enzyme, completing the green Fucci indicator, and generating pBSK Fucci. Next, a BstXI - PacI fragment of pBSK Fucci was cloned into a pPRIG p vector, generating the pPRIFup vector, and finally the PuroR gene was taken from the pSuperRT-Puro vector (a generous gift from Dr Philippe Pognonec) by a BamHI and AsuII digestion, to be cloned in the pPRIFup vector digested by the same enzymes.

#### Cre-HA encoding retrovector

The Cre encoding sequence was taken by digesting the pCAG Cre-GFP by EcoRI and OliI and cloned into the pPRIG HA-c retrovector [40], generating the pPRIG Cre-HA retrovector. The gene encoding the resistance to zeocin was then brought by digesting the pPRIZ vector by PflMI and SapI, and cloned into pPRIG Cre-HA digested by the same enzymes.

### Transduction of EndoC-βH2 cells with ecotropic retrovectors

Ecotropic retrovectors were used to introduce the genes encoding the Fucci indicators in EndoC-βH2 cells. Expression of the receptor for murine leukemia virus ecotropic envelope mCAT1 (mouse Cationic Amino acid Transporter, also known as Slc7a1) is sufficient to endow human cells with permissiveness for transduction with ecotropic retrovectors [41], [42]. Two distinct procedures were followed to express mCAT1 in EndoC-βH2 cells and both of them turned out to be very efficient: i) exposure of EndoC-βH2 cells to mCAT1 gesicles, followed by transduction with an ecotropic retrovector encoding one Fucci indicator together with a selectable marker [43]. This procedure was followed to generate the EndoC-βH2-OFP-GFZ cells in a two-step manner: firstly, EndoC-βH2-cells were exposed to mCAT gesicle, then transduced with a retrovector encoding the orange Fucci indicator together with a selectable marker (resistance to puromycine, in pPRIPu vector). After selection in puromycin (2 µ/ml), the resulting cells (termed EndoC-βH2-OFP) were submitted to a second round of gene transfer through exposure to mCAT vesicles followed by transduction with a retrovector encoding the green Fucci indicator together with another selectable marker (resistance to Zeocin, in pPRIZ vector). This second step generated the EndoC-βH2-OFP-GFZ Fucci cells, which were routinely cultured in in hygromycin (50 µ/ml) (Sigma-Aldrich) and zeocin (50 µ/ml) (Invitrogen) medium (Fig. 1A); ii) stable transduction with a VSVG-pseudotyped retrovector encoding mCAT1 (with a C-terminal HA-flag, hence mCAT-HA) and a selectable marker (Hygromycin resistance, in pPRIHy vector); the resulting cells (termed EndoC-βH2-mCAT-HA) were then submitted to transduction with an ecotropic retrovector encoding the two Fucci indicators (separated by a 2A « self-cleavable » peptide) and another selectable marker (resistance to puromycin, in pPRIPu). This gave rise to the EndoC-βH2-PGF2AOF Fucci cells, which were routinely cultured in hygromycin (50 µ/ml) (Sigma-Aldrich) and puromycin (2 µ/ml) medium (Fig. 1B).

#### Retrovector and mCAT gesicle production

Supernatants containing ecotropic retrovectors were produced by a coransfection of three vectors: CMV-Gag-Pol (2 µg), FBMo-Salf (encoding the ecotropic envelope, 2 µg) and any retroviral vector pPRIG derivative (4 µg) the in HEK293 T cells using GenExtrem 9 (Roche) (24 µl for a 75 cm^2^ flask). After 48 h, the supernatants was collected, filtered (0.45 µ) and either directly used or concentrated onto Amicon Ultra-15 (100 kDa) (Millipore) and stored art −80°C. VSVG pseudotyped retrovectors were produced in the same manner except that the FBMoSalf vector was replaced with a VSVG encoding vector (CMV-VSVG). For the production of mCAT1 containing gesicles, HEK293 T were cotransfected in 75 cm^ 2^ flask using GenExtrem 9 (24 µl) with two vectors, CMV-mCAT1 and CMV-VSVG (4 µg each). After 48 h, the supernatants were collected, filtered (0.45 µ) and either directly used or concentrated and stored as retroviral supernatants.

#### Gene transfer into EndoC-βH2 cells

EndoC-βH2 cells and their derivatives were transduced with either ecotropic or VSVG-pseudotyped retrovectors. Half of the supernatant of HEK293 T retrovector producing cells (6 ml for a 75 cm^2^ flask, 100 µl if concentrated) was added (not diluted if not concentrated) for 4–6 hours to 6×10^6^ EndoC-βH2 cells in presence of polybrene (5 µg/ml) (Sigma-Aldrich) which were then refed with fresh medium for 24 hours. Transduced cells were selected in presence of the appropriate drug(s). After one week, selected cells were routinely cultured in the presence of half of the dose used for selection, except for puromycin which was kept at 2 µg/ml. For transduction using mCAT1 gesicles, 6×10^6^ EndoC-βH2 cells were exposed for 4 hours to unmodified (not diluted) or amicon-concentrated (6 ml or 100 µl, respectively) supernatant containing mCAT1 gesicles. Then, the medium was removed and replaced with a ecotropic retroviral containing supernatant (6 ml not diluted if used directly or 100 µl if concentrated, as described above) again for 4 hours, and finally removed to be replaced with fresh medium. Both the exposure to mCAT1 gesicles and to ecotropic retrovectors were performed in presence of polybrene (5 µ/ml).

### Flow cytometry and FACS sorting

Cells were collected by trypsinisation, fixed in PBS containing 3% methanol-free formaldehyde (Electron Microscopy Sciences) for 10 minutes at room temperature, and analyzed by flow cytometry using a Fortessa cytometer (BD Biosciences). 10,000 events were recorded and doublets were excluded. When Hoechst 33342 staining was performed, cells were permeabilized after fixation (NP40 0.2% in PBS for 10 min) and exposed to Hoechst 33342 (Invitrogen) in PBS (3 µg/ml) for 2 hours before analysis. For EdU (5-Ethynyl-2′-deoxyUridine) incorporation, cells were exposed to EdU (10 µM) for two hours before fixation. EdU was revealed by the Click-it EdU Alexa 647 Flow Cytometry Assay kit (Invitrogen). Cell sorting was performed using a FACSJAZZ cell sorter (BD Biosciences). Spectral parameters were as follows. Hoechst 33342: laser 355 nm, filter 450/50; eGFP and mAG: laser 488 nm, filter 525/50; mKO2: laser 561 nm, filter 585/15; Alexa 647: laser 640 nm, filter 670/30.

### Quantitative RT-PCR

Total RNA from EndoC-βH2 cells and their Fucci derivatives was extracted using Qiagen RNeasy plus microkit. Total RNA was retrotranscribed using the Superscript reagents (Invitrogen) following the instructions of the manufacturer. The RT reaction was diluted 20 fold (final volume: 400 µl) and 5 µl were used for each point in quantitative PCR (Q-PCR) using Sybr Green PCR Master Mix using 7300 Fast real-time PCR system (Applied Biosystems). *n*-Fold changes in the expression of each tested gene were calculated using the ΔΔCt method of relative quantification, using the expression levels of *Cyclophilin A* (*CycA*) mRNA for normalization.

### Time lapse video-microscopy

EndoC-βH2-OFP-GFZ and EndoC-βH2-PGF2AOF cells were seeded on matrigel- and fibronectine- coated µ-Slide 8 well (Ibidi, Biovalley) in the standard medium of EndoC-βH2 cells (see Experimental procedures of the main text). 24 hours later, they were observed using a Nikon videomicroscope TIRF (magnification 40X) and photographed each hour. The overlay of the three images (mAG and mKO2 fluorescences, and white light) is shown.

### Primer sequences

Cre recombinase Forward: CGATGCAACGAGTGATGAGG; Reverse: ACCGGCAAACGGACAGAAGC.

Cyclophilin Forward: ATGGCAAATGCTGGACCCAACA; Reverse: ACATGCTTGCCATCCAACCACT.

Ki67 Forward: AGCACCAGAGGAAATTGTGGAGGA; Reverse: ATGATGACCACGGGTTCGGATGAT.

IAPP Forward: TTGGTGCCATTCTCTCATCTAC; Reverse: CAAGTAATTCAGTGGCTCTCTCT.

Insulin Forward: AGAGGCCATCAAGCAGATCACTGT; Reverse: ACAGGTGTTGGTTCACAAAGGCTG.

Proliferating Cell Nuclear Antigen Forward: ATCCTCAAGAAGGTGTTGGAGGCA; Reverse: ACGAGTCCATGCTCTGCAGGTTTA.

SV40 Large T antigen Forward: TGCCTGGAACGCAGTGAGTTTT; Reverse: AACTCAGCCACAGGTCTGTACCAA.

Cyclin E1: Taqman gene expression assay Hs01026536_m1 was used (Applied Biosystems).

## Supporting Information

Figure S1 **Determination of thresholds for flow cytometry analyses of human Fucci beta cells.** Parental EndoC-βH2 cells and two derived cell lines, termed EndoC-βH2-GF and EndoC-βH2-OF, were fixed and analyzed for flow cytometry as described in Experimental procedure of the main text. The EndoC-βH2-GF and EndoC-βH2-OF cell lines were obtained by transducing EndoC-βH2 cells with a retrovector (pPRIPu [40]) encoding either the green Fucci (mAG-ΔGEMININ) or orange Fucci (mKO2-ΔCDT1) and subsequent selection in puromycine containing medium (2 µg/ml). Doublets were excluded from the analyses. Thresholds of green and orange fluorescence were determined according to the level of autofluorescence generated by parental EndoC-βH2 cells (upper panel), and to the level of orange (mKO2) and green (mAG) fluorescence emitted by EndoC-βH2-OF (middle panel) and EndoC-βH2-GF (lower panel) cells, *i.e*. EndoC-βH2 cells stably transduced with a retrovector encoding mAG-ΔGEMININ or mKO2-ΔCDT1, respectively (in addition with PuroR as selectable marker). These two « single positive » cell lines were used for compensation to avoid any « bleeding » of the green fluorescence in the orange channel, and *vice versa*, when EndoC-βH2-OFP-GFZ or EndoC-βH2-PGF2AOF cells were analyzed. Doublets were excluded from the analyses. The overlay of the Fucci fluorescence of the cells and their distribution within the cell cycle according to staining with Hoechst 33342 is shown (right panels).
(TIF)

Figure S2 **Time lapse videomicroscopy on EndoC-βH2-OFP-GFZ cells: S/G2>M>G1 transition.**
(TIF)

Figure S3 **Time lapse videomicroscopy on EndoC-βH2-PGF2AOF cells: S/G2>M>G1 transition.**
(TIF)

Figure S4 **Time lapse videomicroscopy on EndoC-βH2-OFP-GFZ cells: G1>S transition.**
(TIF)

Text S1 **Supporting text.**
(PDF)
