# Supplementary figures and images for: Human Fucci Pancreatic Beta Cell Lines: New Tools to Study Beta Cell Cycle and Terminal Differentiation
Source: PLoS One. 2014 Sep 26;9(9):e108202. doi: 10.1371/journal.pone.0108202 (PMC4178124; doi:10.1371/journal.pone.0108202)

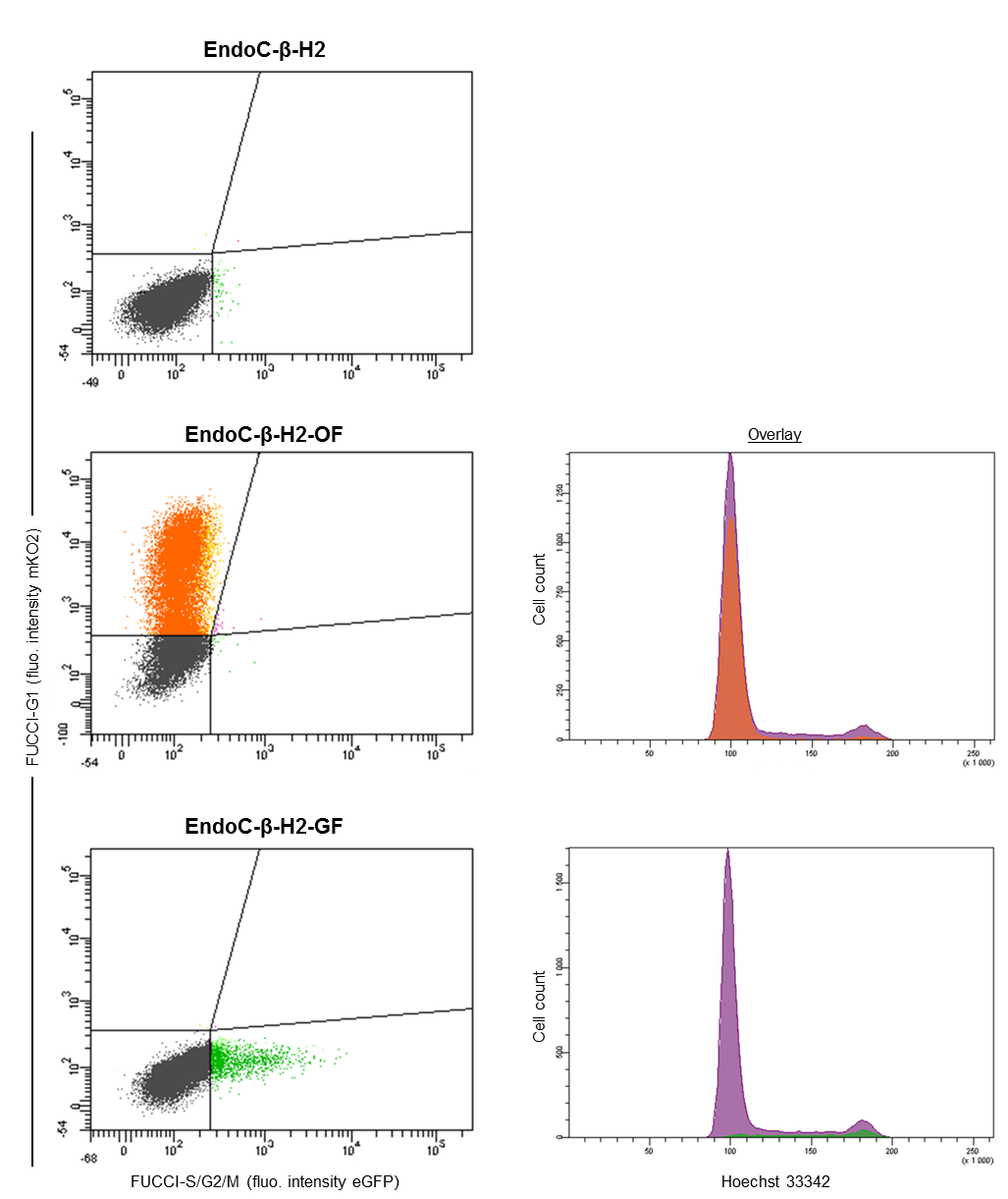

Supplement: Figure S1 — Determination of thresholds for flow cytometry analyses of human Fucci beta cells. Parental EndoC-βH2 cells and two derived cell lines, termed EndoC-βH2-GF and EndoC-βH2-OF, were fixed and analyzed for flow cytometry as described in Experimental procedure of the main text. The EndoC-βH2-GF and EndoC-βH2-OF cell lines were obtained by transducing EndoC-βH2 cells with a retrovector (pPRIPu [40]) encoding either the green Fucci (mAG-ΔGEMININ) or orange Fucci (mKO2-ΔCDT1) and subsequent selection in puromycine containing medium (2 µg/ml). Doublets were excluded from the analyses. Thresholds of green and orange fluorescence were determined according to the level of autofluorescence generated by parental EndoC-βH2 cells (upper panel), and to the level of orange (mKO2) and green (mAG) fluorescence emitted by EndoC-βH2-OF (middle panel) and EndoC-βH2-GF (lower panel) cells, i.e. EndoC-βH2 cells stably transduced with a retrovector encoding mAG-ΔGEMININ or mKO2-ΔCDT1, respectively (in addition with PuroR as selectable marker). These two « single positive » cell lines were used for compensation to avoid any « bleeding » of the green fluorescence in the orange channel, and vice versa, when EndoC-βH2-OFP-GFZ or EndoC-βH2-PGF2AOF cells were analyzed. Doublets were excluded from the analyses. The overlay of the Fucci fluorescence of the cells and their distribution within the cell cycle according to staining with Hoechst 33342 is shown (right panels). (TIF) [file pone.0108202.s001.tif]

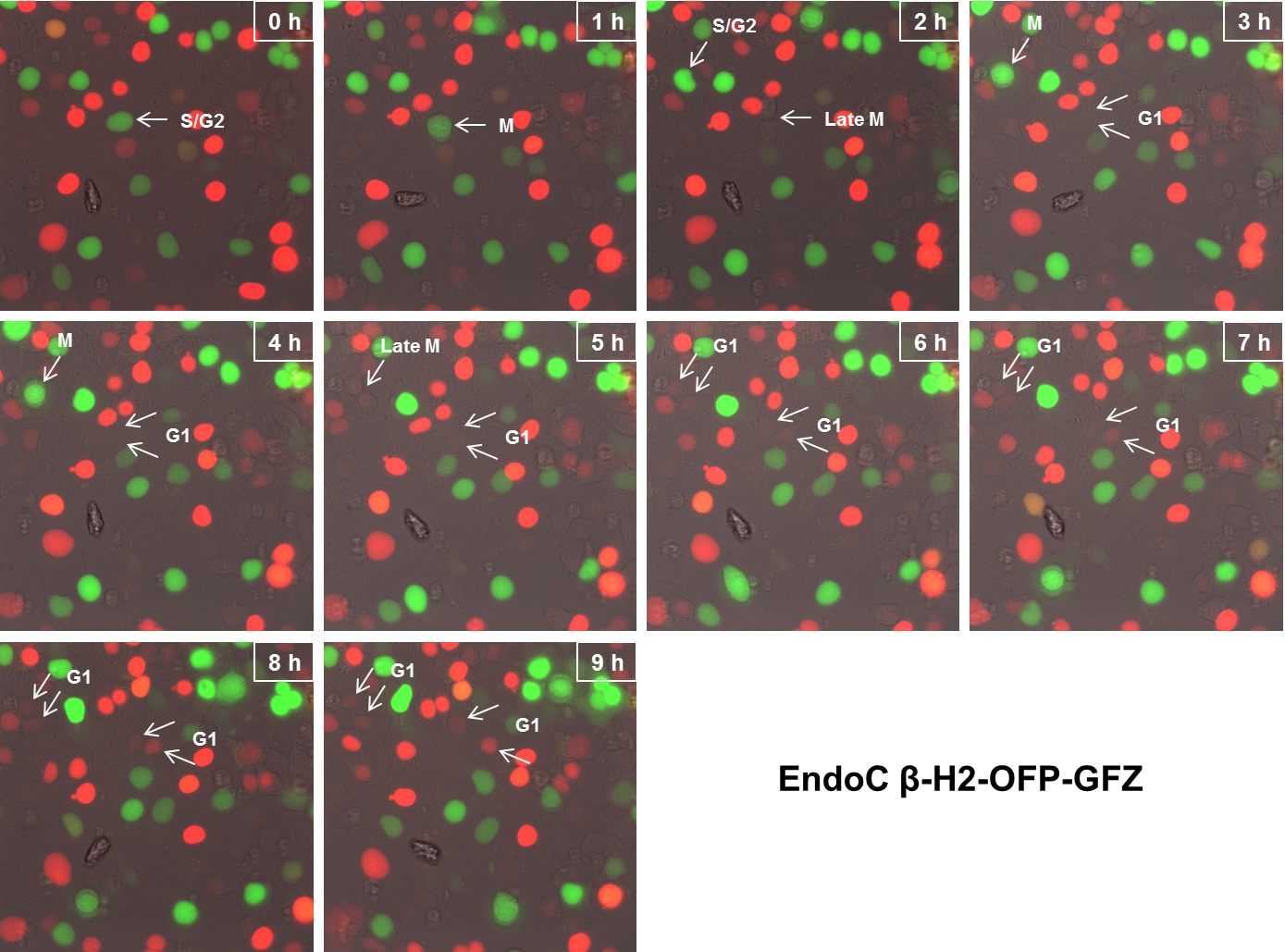

Supplement: Figure S2 — Time lapse videomicroscopy on EndoC-βH2-OFP-GFZ cells: S/G2>M>G1 transition. (TIF) [file pone.0108202.s002.tif]

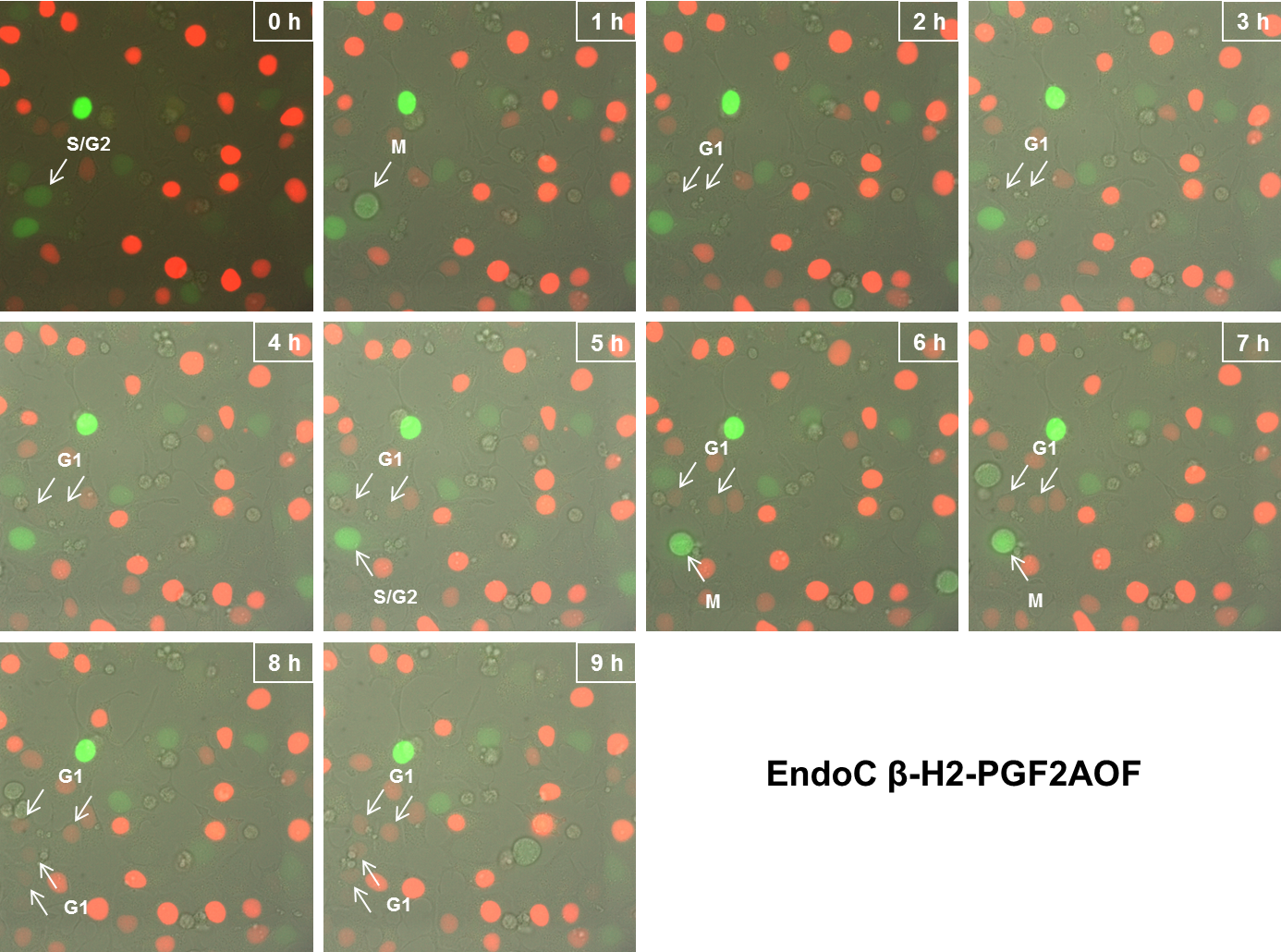

Supplement: Figure S3 — Time lapse videomicroscopy on EndoC-βH2-PGF2AOF cells: S/G2>M>G1 transition. (TIF) [file pone.0108202.s003.tif]

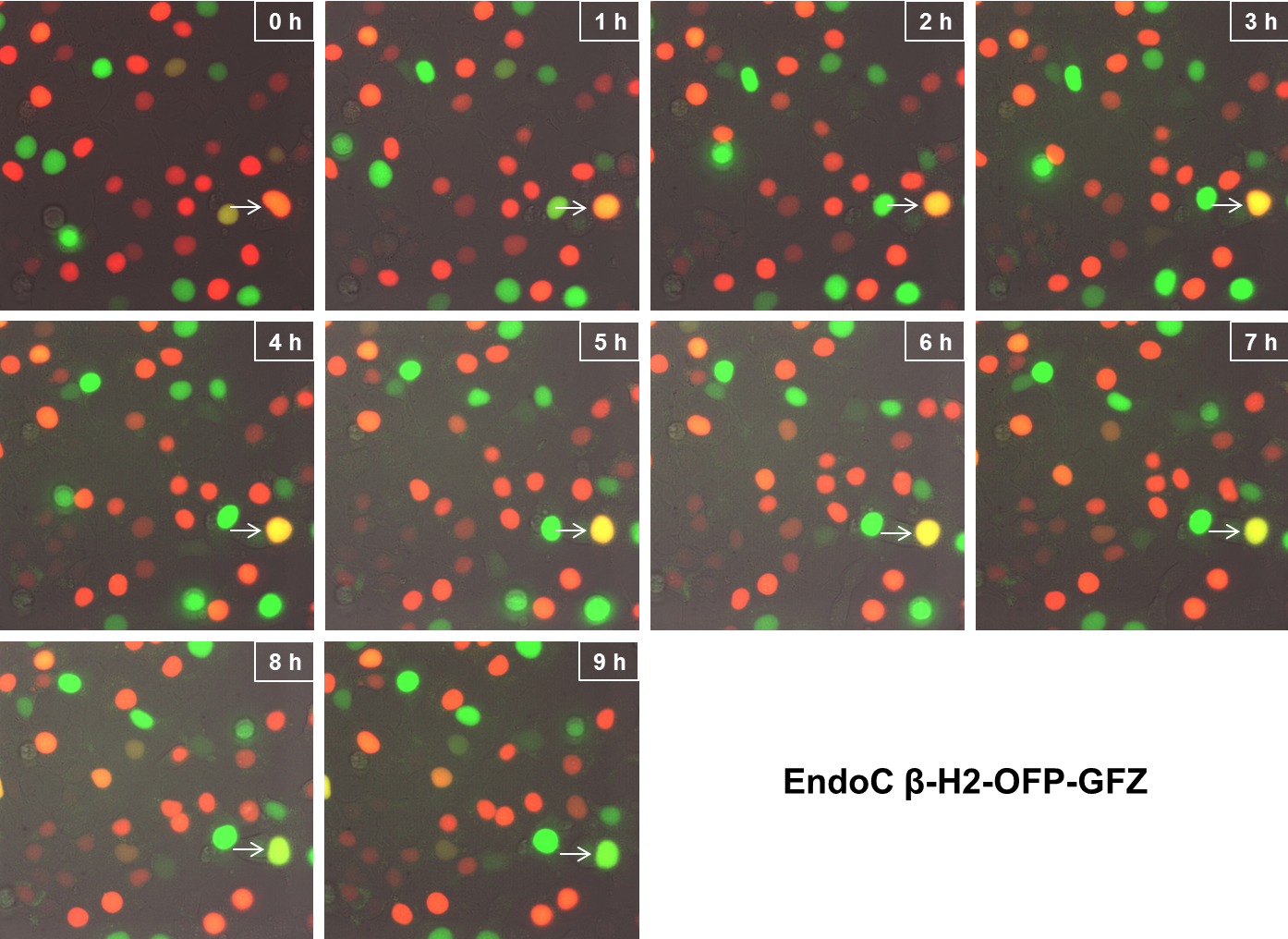

Supplement: Figure S4 — Time lapse videomicroscopy on EndoC-βH2-OFP-GFZ cells: G1>S transition. (TIF) [file pone.0108202.s004.tif]
