# Supplementary material for: Human Fucci Pancreatic Beta Cell Lines: New Tools to Study Beta Cell Cycle and Terminal Differentiation
Source: PLoS One. 2014 Sep 26;9(9):e108202. doi: 10.1371/journal.pone.0108202 (PMC4178124; doi:10.1371/journal.pone.0108202)
Supplement: Text S1 — Supporting text. (PDF) [file pone.0108202.s005.pdf]

Supporting Information

Supplementary Figures

Figure S1. **Determination of thresholds for flow cytometry analyses of human Fucci beta cells.** Thresholds of green and orange fluorescence were determined according to the level of autofluorescence generated by parental EndoC-βH2 cells (upper panel), and to the level of orange (mKO2) and green (mAG) fluorescence emitted by EndoC-βH2-OF (middle panel) and EndoC-βH2-GF (lower panel), *i.e*. EndoC-βH2 cells stably transduced with a retrovector encoding mAG-ΔGEMININ or mKO2-ΔCDT1, respectively (in addition with PuroR as selectable marker). These two « single positive » cell lines were used for compensation to avoid any « bleeding » of the green fluorescence in the orange channel, and *vice versa*, when EndoC-βH2-OFP-GFZ or EndoC-βH2-PGF2AOF were analyzed. Doublets were excluded from the analyses. The overlay of the Fucci fluorescence of the cells and their distribution within the cell cycle according to staining with Hoechst 33342 is shown (right panels).

Figure S2. Time lapse videomicroscopy on EndoC-βH2-OFP-GFZ cells: S/G2>M>G1 transition.

Figure S3. Time lapse videomicroscopy on EndoC-βH2-PGF2AOF cells: S/G2>M>G1 transition.

Figure S4. Time lapse videomicroscopy on EndoC-βH2-OFP-GFZ cells: G1>S transition.

Supplementary methods

Flow cytometry

Parental EndoC-βH2 cells and two derived cell lines, termed EndoC-βH2-GF and EndoC-βH2-OF, were fixed and analyzed for flow cytometry as described in Experimental procedure of the main text. Doublets were excluded from the analyses. The EndoC-βH2-GF and EndoC-βH2-OF were

obtained by transducing EndoC-βH2 cells with a retrovector (pPRIPu) encoding either the green Fucci (mAG-ΔGEMININ) or orange Fucci (mKO2-ΔCDT1) and subsequent selection in puromycine containing medium (2 µ/ml).

Time lapse videomicroscopy

EndoC-βH2-OFP-GFZ and EndoC-βH2-PGF2AOF cells were seeded on matrigel- and fibronectine- coated µ-Slide 8 well (Ibidi, Biovalley) in the standard medium of EndoC-βH2 cells (see Experimental procedures of the main text). 24 hours later, they were observed using a Nikon videomicroscope TIRF (magnification 40X) and photographed each hour. The overlay of the three images (mAG and mKO2 fluorescences, and white light) is shown.
